# Effectiveness of CAD-CAM Milled Versus DMLS Titanium Frameworks for Hybrid Denture Prosthesis: A Narrative Review

**DOI:** 10.3390/jfb15120376

**Published:** 2024-12-12

**Authors:** Yahya Deeban

**Affiliations:** Department of Prosthodontics and Restorative Dentistry, College of Dentistry, Majmaah University, Al Majmaah 11952, Saudi Arabia; y.deeban@mu.edu.sa or yahyadeeban@gmail.com

**Keywords:** CAD-CAM system, dental implants, direct metal laser sintering, dental prosthesis design, hybrid dentures

## Abstract

This narrative review aimed to evaluate the effectiveness of computer-aided design (CAD), computer-aided manufacturing (CAM) milled, and direct metal laser sintering (DMLS) titanium frameworks in hybrid denture prostheses. A structured PICO analysis and a review of ten publications were used to compare titanium frameworks for hybrid dentures made through milling, DMLS, and CAD-CAM milling. Prosthesis success, bone loss, patient satisfaction, framework fit, and biofilm adhesion were among the outcome indicators. The inclusion criteria included comparisons between DMLS and milled titanium frameworks, investigations of hybrid dentures with metal frameworks, and various study designs. The exclusion criteria included reviews, case reports, non-comparative research, and studies unrelated to hybrid dentures. A comprehensive search was performed up to December 2023 across PubMed, PubMed Central, Cochrane Library, Scopus, and Google Scholar, using terms such as CAD-CAM, dental implantation, dental prosthesis, bone loss, damaged maxilla/mandible, implant framework, and bone volume. Ten studies were available for the final analysis. These studies shed light on milled titanium framework’s relative effectiveness and characteristics versus DMLS for implant-supported hybrid dentures. This narrative analysis clarifies the critical roles of the CAD-CAM and DMLS frameworks in implant-supported hybrid dentures. Despite the significant benefits of both of these technologies, it is evident that more investigation is required to identify the optimal framework option for specific clinical scenarios, highlighting the importance of continuing research in this field.

## 1. Introduction

Dentistry uses computer-aided design (CAD) and computer-aided manufacturing (CAM) technology to revolutionize prosthodontics by addressing biocompatibility and aesthetic issues [1,2,3]. CAD-CAM dental prosthesis systems utilize various materials, such as glass ceramics, polycrystalline ceramics, resin composites, and titanium [4]. Due to its strength, durability, and biocompatibility, titanium has become increasingly popular in dentistry, particularly in hybrid denture prosthesis [5,6]. The emphasis on precision and customization in CAD-CAM, particularly when working with titanium, highlights positive patient outcomes [5]. Recent evaluations have emphasized the precision and customization in CAD-CAM and 3D-printed dentures [4,7]. The materials and production methods used greatly impact the effectiveness of dental prostheses, particularly in complex cases such as natural teeth and implants [8]. This narrative review examines and contrasts the methods for creating titanium frameworks for hybrid denture prostheses using direct metal laser sintering (DMLS) and CAD-CAM milling. Fit accuracy, mechanical characteristics, aesthetics, and clinical performance are among the factors evaluated.

***Fit accuracy and precision:*** A fit, precise framework is critical for an effective implant-supported dental prosthesis because it ensures stability, reduces problems, and improves patient comfort [9]. Experts highly praise the remarkable accuracy and dependability of CAD-CAM technology in framework construction, as it plays a crucial role in implant-supported hybrid denture prosthesis [10]. Numerous investigations have demonstrated the low marginal defects and excellent fit precision of CAD-CAM machined titanium frameworks [11,12,13]. With the help of virtual planning and digital impressions, CAD-CAM produces digital precision that melds perfectly with the patient’s implants and dentition [14]. Revilla-León et al. [15] have pointed out that DMLS technology, on the other hand, might show larger fit disparities. This could be due to the layering process, which introduces small imperfections.

***Mechanical properties:*** The mechanical properties of titanium frameworks and fit precision greatly impact the long-term survival of hybrid denture prosthesis. The mechanical characteristics of titanium, which is well known for its excellent strength-to-weight ratio and resistance to corrosion in dental applications, differ depending on the manufacturing method [5,16]. Made from solid blocks, CAD-CAM-milled titanium frameworks have an isotropic and homogenous structure that provides consistent mechanical properties for increased strength and stability [11]. DMLS, on the other hand, uses lasers to fuse titanium particles layer by layer. This creates a multilayer structure that raises concerns about its long-term performance and changing mechanical properties, particularly in high-stress areas like the mouth [15,17].

***Aesthetic outcomes:*** Aesthetic outcomes, alongside fit and mechanical properties, are crucial for patient satisfaction with hybrid denture prostheses. Patients also appreciate a natural appearance, in addition to the utility of treatments [18]. CAD-CAM-machined titanium frameworks accurately reproduce color and shape, ensuring a perfect fit with the patient’s natural teeth, surpassing expectations in terms of appearance [19]. In addition to improving the prosthesis’ overall appearance, the technique makes it easier to use aesthetically pleasing materials like ceramic veneers [19]. Compared to CAD-CAM-milled frames, DMLS technology provides greater design flexibility; nonetheless, it may also require more time and resources to achieve an equally beautiful result. It has been reported that the layers in the manufacture of DMLS can complicate achieving a smooth surface and often necessitate post-processing techniques such as veneering or polishing [15].

***Clinical performance and longevity:*** Hybrid denture prostheses require long-term clinical performance and longevity, which is essential for preserving long-term dental health and patient satisfaction [20,21]. Extensive investigation has demonstrated the therapeutic effectiveness of CAD-CAM-machined titanium frames. Published literature demonstrated outstanding ten-year survival rates for CAD-CAM milled frameworks supporting implants in the edentulous jaw, highlighting the clinical prowess and stability attributable to exact fit and mechanical attributes [22,23,24]. A CAD-CAM subtype of titanium frames with laser welding also has an exceptional lifetime due to its inherent strength and precise fit [24,25]. On the other hand, given possible difficulties in terms of fit accuracy and differences in mechanical properties, DMLS titanium frameworks, despite their design advantages, require more research to fully understand their therapeutic efficacy [15]. The CAD-CAM technique is also equally effective for producing removable partial dentures (RPDs) with a clinically acceptable fit. It provides accurate data acquisition and processing, and the milling techniques used in the CAM process are similar to conventional fabrication techniques. However, further refinement is required to achieve comparable outcomes. Conventional techniques such as flask-pack-pressing or injection molding are used for fabricating dentures, while CAD-CAM methods use additive or subtractive processes [4,7]. The precision of scanning, the numerical control program’s 3D model conversion, and the milling machine’s accuracy capabilities can affect the outcomes of fabricating RPD frameworks. Previous studies have shown that optical scanning and virtual design can fabricate RPDs with clasps; nevertheless, transforming CAD data into CAM processing introduces imperfections. Incomplete data sets and the combination of two different designer tools can generate software errors. Future studies should focus on optimizing technical parameters during the fabrication process to improve the accuracy of RPD fabrications [22,23].

The development of DMLS and CAD-CAM technology has had a significant impact on the fit, mechanical characteristics, aesthetics, and clinical performance of titanium framework production for hybrid denture prostheses [15,21]. Clinical data firmly back up the potential aesthetic appeal, consistency in mechanical properties, fit, longevity, and precision of CAD-CAM-machined titanium frames [4]. Nevertheless, further research is necessary to fully comprehend the variety of mechanical properties and fit accuracy offered by DMLS titanium frameworks. Prioritizing patient needs, aesthetics, and clinical knowledge when deciding between CAD-CAM milled and DMLS titanium frameworks will ensure overall patient health and prosthesis success. The differences between both frameworks based on specific characteristics can be enumerated as summarized in Table 1. The key differences in the efficacy and general properties of both these materials are summarized in Table 2. It is very important to establish the effectiveness of CAD-CAM milled and DMLS titanium frameworks for hybrid denture prostheses. Therefore, the present narrative review aimed at evaluating and comparing CAD-CAM complete dentures (CDs) with conventionally fabricated CDs in terms of true compatibility, mechanical properties, surface characteristics, color stability, time-cost analysis, and clinical outcomes.

## 2. Methodology

**Research question:**

***Population:*** The target population for this review included healthy adults who were entirely edentulous and had enough bone support for the creation of implant-supported hybrid dentures.

***Intervention:*** Hybrid dentures with a titanium metal framework using computer-aided design (CAD) and computer-aided manufacturing (CAM).

***Comparison:*** Hybrid dentures supported by direct metal laser sintering (DMLS).

Outcome measures:

In this study, the following outcome metrics were evaluated:

- The overall success rate of the prosthesis.
- Loss of bone surrounding the implants’ supporting structures.
- Patient contentment.
- The framework’s vertical fit.
- Biofilm adhesion to the structure.

**Inclusion criteria:**

- Research on hybrid dentures that use metal for their framework.
- Articles comparing the two types of titanium used as the framework for hybrid dentures: DMLS titanium and milled titanium.
- Study designs that include in vitro research, animal studies, prospective or retrospective cohort studies, randomized controlled trials, and non-randomized controlled trials.

**Exclusion criteria:**

- Studies not focusing on hybrid dentures
- Absence of metal framework material
- Non-comparative studies
- Review articles
- Case reports

**Search strategy:**

To establish a comprehensive list of relevant studies that have been subjected to peer review, a comprehensive search of the published literature up to December 2023 was conducted. The following databases were systematically searched: PubMed, PubMed Central, Cochrane Library, Scopus, and Google Scholar. The search strategy was as follows: “*CAD CAM*”, “*Computer-Aided Design Computer-Aided Manufacture*”, “*dental implantation*” *OR* “*implant-supported*” *OR* “*oral rehabilitation*” *OR* “*dental implants*”, “*dental prosthesis*”, “*denture framework*”, “*alveolar bone loss*”, “*resorbed maxilla*” *OR* “*atrophic maxilla*”, “*resorbed mandible*” *OR* “*atrophic mandible*”, “*compromised maxilla*”, “*compromised mandible*”, “*implant framework*”, “*maxillary neoplasms*” *OR* “*maxillectomy*” *OR* “*reconstruction*”, “*mandibulectomy*”, “*bone volume*” *OR* “*bone quantity.*” In contrast to a systematic review, this one lacks a clear hypothesis and instead covers a broad discussion of the subject. The citation lists of the included references were then assessed, and a manual search was also carried out in an attempt to locate further papers. A single examiner carried out the data search and analysis for this in-depth narrative assessment.

## 3. Results

An initial search using Google Scholar, Cochrane Library, PubMed, PubMed Central, and Scopus produced 236 results. A more targeted PubMed search, which considered abstracts and titles, yielded 88 publications. A total of 29 articles were removed upon going through titles, and 49 articles that satisfied the inclusion and outcome measure requirements were included. After carefully reviewing the abstract and title, 39 articles were removed from the final list. The final review contained 10 papers that passed this rigorous screening process. The table provides a concise overview of the methodical search strategy and screening procedure, highlighting the cautious selection of pertinent articles for an in-depth narrative assessment. Based on 10 relevant studies conducted over several years, Table 3 summarizes a holistic description of the basic study focus, methodology, and primary findings for the included studies. It also provides a concise summary of the 10 studies in this narrative review that examine various techniques for fabricating dental frameworks in prosthetic dentistry. Among them, five studies were in vitro studies [12,15,27,43,44], one was a prospective clinical trial [42], one was a case series [45], and the other three were comparative studies [11,22,24]. A Swedish study reported the outcomes in 155 patients after 10 years [22] and 15 years [24]. Jemt et al. conducted a prospective study using titanium CNC-milled frameworks for missing teeth jaw implants, which showed how important accuracy is for good results [42]. Ortorp et al. reported a 10-year clinical outcome study on laser-welded titanium frameworks in edentulous mandibles, demonstrating their durability and efficacy [22]. A 15-year follow-up research study evaluated early laser-welded titanium frames, providing insights into their design and operation [24]. An additional 10-year study assessed the efficacy of CNC-milled titanium frameworks in the edentulous jaw [11]. An in vitro study from Japan investigated the use of selective laser melting technology to create titanium alloy frameworks for full dentures [27]. The in vitro investigation performed by Arnold et al. [12] concentrated on CAD-CAM accuracy for detachable partial dentures. Ciocca et al. [43] compared metal framework fabrication techniques, and Revilla-León et al. [15] studied full-arch titanium frameworks using additive manufacturing. Patients with implant-supported prostheses could benefit from using CNC-milled titanium frameworks instead of gold-alloy castings [11,12]. Milling techniques enabled the fabrication of RPDs with comparable or better fits compared to the LWT. Nevertheless, rapid prototyping techniques showed distinct fitting irregularities [22]. The titanium frameworks that were tested for a full-arch implant-supported prosthesis made with either SLM or EBM additive technology had differences between the implants and the prosthesis that were clinically acceptable. The current treatment modality could maintain predictable overall long-term results [27]. Fractures of the metal frames and re-made prostheses were more common in the test group, and gold alloy frameworks tended to work better than welded titanium frameworks over the past 15 years [24]. All observed technologies enable acceptable results, with DMLS and machine milling outperforming conventional technologies, except in terms of cost. The suggested modification of the conventional casting process enables better accuracy at a lower cost [45].

Table 4 and Table 5 summarize the key findings from a few studies using CAD-CAM and DMLS techniques for hybrid prostheses, providing insight into the accuracy and usefulness of computer-aided design and manufacture in prosthodontics. Arnold et al. evaluated digitally manufactured removable partial dentures focused on precision measures using CAD-CAM [12]. Vigolo et al. [46], Passia et al. [47], and Berrendero et al. [48] focused on long-term clinical outcomes on zirconium oxide-based, ceramic, fixed denture prostheses. However, these studies were excluded from the final analysis. These studies highlight the growing importance of CAD-CAM in prosthetic dentistry, suggesting improved precision and longevity. Table 4 summarizes perspectives from CAD-CAM research on long-term prosthetic dental assessment.

Table 5 summarizes the perspectives of DMLS research on promoting hybrid prosthetics. Penchev [45] looked at metal frameworks for dentures, Revilla-León et al. [15] compared different types of additive manufacturing, and Kanazawa et al. [27] studied DMLS and discussed the use of titanium alloy frameworks. These studies demonstrate the importance of additive manufacturing in dental prosthesis and emphasize the need for a thorough investigation into the DMLS compared to established and cutting-edge metal framework fabrication methods.

## 4. Discussion

The conventional design of a hybrid prosthesis to replace the missing teeth often involves coating a metal substructure with acrylic resin [9]. Numerous studies have reported on the construction of titanium frames using computer aids. These studies include hybrid dentures [11,12,15,22,24,27,42,43,44,45] and fixed prostheses [46,47,48,49,50]. Casting was the usual approach for manufacturing metal frameworks; however, this method was prone to problems, including porosity and distortion, due to the expansion and contraction that occurred during casting [51]. Casting was the method that was used, and the advancement of CAD-CAM in dentistry means it is now possible to create prostheses with greater accuracy, homogeneity, and cost-effectiveness [12,52,53,54]. The present narrative review explored several hybrid bar production techniques, with a special emphasis on milled and printed titanium bars [5]. The review found 10 papers that described the use and characteristics of CAD-CAM technology and DMLS frameworks for full-arch prostheses. This decision clarifies the potential benefits of a specific framework manufacturing method for prostheses. Long-term clinical trials are surprisingly difficult, despite the ubiquitous usage of CAD-CAM technology for titanium hybrid bars [55]. Nonetheless, various studies reported on the metal frameworks for hybrid dentures, along with clinical follow-ups. However, few studies have observed the strength of titanium frameworks crafted using various techniques and their impact on implants and surrounding bone strain [44,56]. In these investigations, cumulative survival rates (CSR) and bone loss around implants were the most frequently studied outcomes. Interestingly, there have been few attempts to quantify patient-centered outcomes and to compile information on the various types of prosthetic failures reported [41]. With the introduction of modern manufacturing technologies, particularly CAD-CAM and DMLS, hybrid prostheses in implant dentistry have undergone a significant transformation.

The use of CAD-CAM technologies has led to widespread use in the field of dental implantology [11]. These frameworks have shown remarkable cumulative survival rates (CSRs) when combined with gold alloys; for example, CNC-milled titanium bars have a CSR of 95.6% [24]. Extended follow-up duration, however, has shown some prosthesis problems, such as implant loss, framework fractures, and the need for revisions, particularly in smokers. Nevertheless, they still showed a higher incidence of veneer fractures, highlighting the need for ongoing improvement [10,14,21]. CNC-milled titanium and gold alloy frameworks also showed fewer fractures than conventional laser-welded titanium frameworks [25]. Direct metal laser sintering represents a promising fabrication strategy, offering distinct advantages compared to traditional methods. Recent studies have highlighted its superior hardness in comparison to cast titanium [27,48,57]. The DMLS frameworks demonstrated impressive precision while maintaining the integrity of the structure [6,7,58]. They achieve this by addressing casting challenges, such as gaps resulting from shrinkage and the occurrence of pores. Comparing various additive manufacturing methods, like selective laser melting (SLM) and electron beam melting (EBM), has shown that, for full-arch implant-supported prostheses, differences between the implant and the prosthesis are clinically acceptable [14,34]. The choice between CAD-CAM and DMLS technologies is a crucial decision in the world of hybrid prostheses. With its accuracy and widespread application, CAD-CAM has produced noteworthy CSR outcomes, but it also presents with veneer fractures over time [10,11,21,25]. Nevertheless, DMLS has exceptional hardness, precision, and lack of porosity, making it an appealing area for investigation in dental implantology. CAD-CAM systems have demonstrated clinical efficacy, characterized by reduced complication rates and elevated patient satisfaction. The present narrative research also demonstrated the versatility of DMLS in producing complex prosthetic designs. These findings highlight the importance of evaluating prosthetic complexity and patient-specific attributes when selecting the optimal manufacturing method, be it CAD-CAM or DMLS, to achieve superior outcomes in hybrid prostheses. Consequently, the decision between CAD-CAM and DMLS technologies for hybrid prostheses is intricate and contingent upon various variables, including the prosthesis’s complexity and patient-specific considerations [59,60,61,62,63]. Dental practitioners can enhance outcomes by meticulously employing these production procedures, which integrate precision, aesthetics, durability, and patient satisfaction to deliver optimal care in hybrid prostheses [61,62,63]. The narrative reviews only offer interpretations that are open to critique and will vary depending on the author team or context of the review. The current review is narrative in nature, and it does not provide an evidence-based synthesis for specific research questions, nor does it offer definitive practice guidelines. These were considered limitations of the present review.

## 5. Conclusions

The narrative review identified ten studies; among them, five were in vitro, and three were comparative studies, one study was a prospective clinical trial, and the other was a case series. All these published studies observed the effectiveness of CAD-CAM-milled and DMLS titanium frameworks for hybrid denture prostheses. These techniques offer advantages such as fewer patient appointments, reduced clinical time, and digital archiving of completed prostheses, and they also reduce manufacturing costs. However, these hybrid dentures have improved mechanical and surface properties and are not inferior to conventional dentures. There is a need to conduct further research to ascertain whether hybrid denture prostheses can utilize either framework.

## Figures and Tables

**Table 1:** Comparison of CAD/CAM and DMLS Titanium Framework Characteristics for Dental Implant Prostheses.

Characteristics	CAD/CAM Framework	DMLS Titanium Framework
Fabrication method	The prosthesis is developed digitally using computer-aided design (CAD) and precision-machined using computer-aided manufacture (CAM). This technique makes complex customization and design possible [12,26].	DMLS employs a laser to melt and fuse titanium powder layer by layer to form intricate shapes. Complex framework designs work nicely with it [15,27].
Precision of fit	The great precision and accuracy of CAD-CAM technologies frequently result in low fit discrepancies [16,24]. For implant abutments, the computerized design ensures a snug fit [12].	DMLS titanium frameworks exhibit little manufacturing distortion and excellent fit accuracy [15,27].
Manufacturing material	The most often utilized materials for CAD/CAM frameworks are titanium, cobalt-chromium, and zirconia. Known for their strength and biocompatibility, these materials are highly durable [12,28,29].	Typically, DMLS frameworks are made of titanium alloys due to their superior mechanical qualities and biocompatibility [27].
Prosthetic options	Prosthetic options enabled by CAD/CAM technology include implant-supported restorations, partial dentures, and full-arch prosthesis [7,30].	DMLS titanium frameworks are especially well-suited for implant-supported prosthesis, they are a preferred material for hybrid dentures [15,27,31].
Framework distortion	The little deformation caused by CAD-CAM technologies during the milling process ensures a precise and stable fit [4,26].	Accurate framework production is made possible by DMLS technology’s minimized distortion due to its layer-by-layer fusing process [12].
Clinical use	The extensive use of CAD-CAM technology in implant dentistry makes it possible to manufacture precise and effective prosthesis [6,29,32].	DMLS is used for implant-supported prosthesis, especially when complex geometries or extreme precision are needed [31,33].
Marginal fit	CAD-CAM frameworks are known for their excellent marginal fit, which enhances patient comfort and long-term prosthesis stability [34].	DMLS titanium frameworks have improved marginal fit, the implant-abutment contact is well sealed [12].
Material biocompatibility	Titanium and cobalt-chromium are used in CAD-CAM systems because of their biocompatibility and body-friendly nature [5,28,35].	Since the titanium alloys utilized in DMLS are biocompatible, they can be employed to create implant prosthesis [12,36,37].
Clinical outcomes	CAD-CAM technology has long-term viability and cosmetic advantages have been connected to better treatment outcomes [29,32,38].	Positive treatment results, excellent long-term performance, and patient satisfaction have been observed with DMLS titanium frameworks [31,33].

**Table 2:** Key Differences between CAD-CAM milled and DMLS Titanium Frameworks for metal prostheses based on general properties.

Factors and Properties	CAD-CAM Framework	DMLS Titanium Framework
Material characteristic	Titanium alloy is often used to make CAD-CAM frameworks [12].	DMLS Titanium frames are another product made of titanium alloy [39].
Mechanical properties	Robustness and longevity are ensured by superior mechanical properties in CAD-CAM frameworks [39].	DMLS Titanium frameworks have exceptional mechanical properties that provide strength and longevity [39].
Surface characteristics	CAD-CAM frameworks’ smooth surface finishes can enhance comfort and appearance [27].	DMLS Titanium frameworks’ flawless surface finish greatly improves patient comfort and aesthetics [40].
Fabrication process	CAD-CAM frames are created using computer-aided design and milling [41].	DMLS Titanium frameworks are produced using 3D printing technology, specifically direct metal laser sintering [27].
Precision fit	CAD-CAM frameworks are highly precise and a perfect fit [42].	DMLS Titanium frames offer great precision and fit as well [24].
Color stability	CAD-CAM frameworks demonstrate color constancy over time [24].	DMLS Titanium frameworks indicate robust color stability as well [29].
Time and cost efficiency	Most people agree that CAD-CAM frameworks allow for faster manufacturing and potentially lower prices [43].	DMLS Titanium frameworks offer affordable, rapid manufacture [31].
Clinical and patient outcomes	It has been shown that clinical outcomes utilizing CAD-CAM frameworks are good [42].	Additionally displaying encouraging clinical outcomes are DMLS Titanium frameworks [33].
Research evidence	There is substantial research to support the use of CAD-CAM frameworks [10].	DMLS Titanium frameworks are being supported by an increasing amount of research, and the results are promising [23].
Clinical longevity	Clinical success with CAD-CAM frameworks has been shown to last over time [41].	Clinical longevity is a promising feature of DMLS Titanium frameworks [32].
Available clinical cases	CAD-CAM frameworks are commonly used in implant-supported fixed and removable dental prostheses, according to Cotton et al. [39].	DMLS Titanium frames are increasingly being used in implant-supported dental prosthesis [43].
Material cost	Costs for CAD/CAM frameworks vary depending on titanium pricing [39].	The price of titanium affects the variable expenses of the framework [39].
Potential limitations	Complex designs may have constraints depending on the milling equipment [10,42].	Limited by construction volume and perhaps requiring Post processing [12,27].
Additional considerations	Possibility of personalization and modification [10,42].	Geometrical complexity is possible [12,27].

**Table 3:** The details of the studies included in the review.

Author/Year	Research Emphasis/Type	Study Type	Principal Findings	Outcomes
Ortorp et al. (2012) [11]	CNC-milled titanium frameworks	Comparative study	Performed a 10-year clinical trial to gather data on the relative efficacy of titanium frameworks of 126 patients with CNC milling and implants in the edentulous jaw.	The efficiency of titanium frameworks machined by CNC in edentulous jaw implants.
Arnold et al. (2018) [12]	CAD-CAM- fabricated removable partial dentures	In vitro study	Evaluated the accuracy of removable partial dentures manufactured with CAD-CAM technology employing digital technology.	Accuracy of detachable partial dentures made with CAD-CAM technology.
Revilla-León et al. (2018) [15]	Titanium frameworks via selective laser melting	In vitro study	To examine the variations in titanium frameworks for entire arches produced by additive manufacturing methods including selective laser melting and electron beam melting.	Distinction between full-arch titanium frameworks produced using selective laser melting and electron beam melting methods in additive manufacturing.
Ortorp et al. (2006) [22]	Laser-welded titanium frameworks	Comparative study	The 10-year longitudinal study provided significant insights into the long-term clinical outcomes of laser-welded titanium frameworks supported by implants in edentulous mandibles. The study provided insights into the clinical outcomes of laser-welded titanium frameworks in implant-supported edentulous mandibles for 10 years.	Both groups demonstrated low complications and comparable clinical and radiological performance, suggesting CNC-milled titanium frameworks as a viable alternative to gold-alloy castings for implant-supported prostheses in the edentulous jaw.
Ortorp et al. (2009) [24]	Early laser-welded titanium frameworks Follow-up study of Ortorp et al. (2006) [22]	Comparative study	Over 15 years, the functionality and longevity of the first titanium frameworks made by laser welding in the edentulous mandible were assessed.	Early titanium frameworks fused by laser in an edentulous mandible for comparison.
Kanazawa et al. (2014) [27]	Selective laser melting for titanium alloy	In vitro study	The feasibility of fabricating titanium alloy frames for complete dentures using selective laser melting technology.	The process of creating titanium alloy frames for complete dentures involves selective laser melting.
Jemt et al. (1999) [42]	CNC-milled titanium frameworks	Prospective clinical trial	The precision of titanium frameworks that were CNC-milled for the treatment of edentulous jaw implants.	Precision of titanium frameworks for edentulous jaw implants that are CNC-milled.
Ciocca et al. (2019) [43]	Comparison between milling and hybrid	In vitro study	Compared the processes used in an in vitro study to produce metal frameworks for full-arch dental restorations on implants, with a particular emphasis on milling and a novel hybrid technology.	Comparing the production of metal frameworks for full-arch dental restorations on implants using milling and a novel hybrid method.
Mangano et al. (2020) [44]	Custom-made 3D printed subperiosteal titanium implants	Case series	A series of case studies on bespoke subperiosteal titanium implants manufactured via 3D printing for elderly patients’ prosthetic restoration of their atrophic posterior mandibles.	Subperiosteal titanium implants were created using 3D printing for a prosthetic replacement.
Penchev (2021) [45]	In vitro comparative study of metal denture framework technologies	In vitro study	Comparative in vitro research of machine milling, hybrid technology, and direct metal laser sintering as methods for fabricating metal denture frameworks.	Technology comparison for the manufacturing of metal denture frameworks.

**Table 4:** Perspectives from CAD/CAM Research on Long-Term Prosthetic Dental Assessment.

Author/Year	CAD-CAM Milled Outcome Measures	Description
Arnold et al. (2018) [12]	Evaluated the accuracy of removable partial dentures manufactured with CAD-CAM technology employing digital technology.	Assessed the accuracy of removable partial dentures produced using CAD-CAM technology. Digital technology was used to perform a thorough evaluation of the dentures made using CAD/CAM technology. The information provided lacked specificity concerning the precision measurements and results.

**Table 5:** Perspectives from DMLS Research in Promoting Hybrid prosthetics.

Author/Year	DMLS Outcome Measures	Description
Revilla-León et al. (2018) [15]	Compared variations in full-arch titanium frameworks made with selective laser melting and electron beam melting methods of additive manufacturing.	- To comprehend the variations in full-arch titanium frameworks made by additive manufacturing, such as selective laser melting (SLM), the study conducted an in vitro experiment. - The main focus was comparing frameworks made with various additive manufacturing techniques, as it offered insights into their differences. - The material that is now accessible does not specifically provide specifics about the variations that have been discovered and their ramifications.2415
Kanazawa et al. (2014) [27]	Investigated the feasibility of fabricating titanium alloy frameworks for complete dentures using selective laser melting technology.	- The study looked into the feasibility of creating titanium alloy frameworks, especially for complete dentures utilizing selective laser melting (SLM) technology. - The main goal was to investigate how the DMLS approach may improve the manufacturing process for complete denture frameworks. - The information supplied lacks specifics regarding DMLS’s achievements and difficulties in developing titanium alloy frameworks.
Penchev (2021) [45]	Performed comparative in vitro research of machine milling, hybrid technology, and direct metal laser sintering as methods for fabricating metal denture frameworks.	- In vitro comparative examination of different metal denture framework production technologies, with a particular emphasis on DMLS. - DMLS was evaluated in comparison to other technologies, revealing its relative benefits and drawbacks. - The material provided lacks specifics about how DMLS performs in terms of results when compared to other technologies.

## Data Availability

Not applicable.
